# Wavelet decomposition analysis in the two-flash multifocal ERG in early glaucoma: a comparison to ganglion cell analysis and visual field

**DOI:** 10.1007/s10633-017-9593-y

**Published:** 2017-06-07

**Authors:** Livia M Brandao, Matthias Monhart, Andreas Schötzau, Anna A Ledolter, Anja M Palmowski-Wolfe

**Affiliations:** 1 Department of Ophthalmology, Basel University Hospital, Basel, BS Switzerland; 2 Carl Zeiss Meditec, Feldbach, Switzerland; 3 Department of Ophthalmology, Medical University of Vienna, Vienna, Austria; 4 Universitätsspital Basel Augenklinik, Mittlere Strasse 91, 4031 Basel, Switzerland

**Keywords:** Discrete wavelet analysis, Glaucoma, Multifocal electroretinogram, Optical coherence tomography, Ganglion cell–inner plexiform layer

## Abstract

**Purpose:**

To further improve analysis of the two-flash multifocal electroretinogram (2F-mfERG) in glaucoma in regard to structure–function analysis, using discrete wavelet transform (DWT) analysis.

**Methods:**

Sixty subjects [35 controls and 25 primary open-angle glaucoma (POAG)] underwent 2F-mfERG. Responses were analyzed with the DWT. The DWT level that could best separate POAG from controls was compared to the root-mean-square (RMS) calculations previously used in the analysis of the 2F-mfERG. In a subgroup analysis, structure–function correlation was assessed between DWT, optical coherence tomography and automated perimetry (mf103 customized pattern) for the central 15°.

**Results:**

Frequency level 4 of the wavelet variance analysis (144 Hz, WVA-144) was most sensitive (*p* < 0.003). It correlated positively with RMS but had a better AUC. Positive relations were found between visual field, WVA-144 and GCIPL thickness. The highest predictive factor for glaucoma diagnostic was seen in the GCIPL, but this improved further by adding the mean sensitivity and WVA-144.

**Conclusions:**

mfERG using WVA analysis improves glaucoma diagnosis, especially when combined with GCIPL and MS.

## Introduction

Early diagnosis in glaucoma is still a challenge. While optical coherence tomography (OCT) technology detects ganglion cell loss before a defect is visualized in the standard automated perimetry (SAP) in some patients [1], in others the SAP defect appears before any ganglion cell layer thinning is detected. Visual electrophysiology offers additional noninvasive tests and provides more objective measures to monitor retinal cell activity.

The multifocal electroretinogram (mfERG, two-flash pattern) technology which allows topographic examination of retinal function is an objective method shown to be sensitive in glaucoma [2–8]. Also, it complements visual field information and may be more sensitive in some patients, especially in cases where defects are seen in OCT but not yet in the visual field (poster ARVO 2014: IOVS. 2014; 55(13):975).

The entire process of retinal visual processing involves the phototransduction cascade with different groups of cells and circuits from the photoreceptors to the ganglion cells. Thus, electrical signals produced by different biological structures contribute to the retinal response of the mfERG that is recorded from the cornea [8–12]. In the standard mfERG, amplitude and implicit time are often analyzed [13]. In the two-flash mfERG, the response is smaller and there are more adaptive influences interfering with traditional analysis [7]. Due to the complexity of the two-flash mfERG waveform, disease-specific local variations may be difficult to identify. The responses from vertical and horizontal pathways are not uniform in time. Therefore, individual wave components, important in, e.g., glaucoma, may be hidden inside the whole summed sequence of activity recorded over a period of time, under a certain stimulus.

Applying mathematical methods, which work as a “filter” in order to identify “hidden characteristics” inside the electrical signal, have been proposed to optimize analysis of electroretinogram (ERG) responses. Bach and Meigen [14] analyzed the application of discrete fourier transform (DFT) in steady-state evoked potentials (i.e., pattern ERG, VEP). Although its application demonstrated good results, DFT has not gained wide acceptance in the analysis of the mfERG [15, 16].

Wavelet analysis, which takes both frequency and time into consideration, is not a new topic in biomedical signals. Its use has been increasingly studied and applied either to improve the interpretation of the biosignal itself (ECG [17], EMG [18]) or to exclude interference between signals [19]. In electrophysiology of vision, this method has been introduced a decade ago [20, 21]. It has been used to analyze the photopic negative response in the full-field ERG of healthy subjects [22]. Barraco et al. [23] compared different analytical approaches (including application of the continuous wavelet transform, CWT) to analyze the *a*-wave of the full-field ERG, demonstrating consistently altered photoreceptor behavior in various diseases. Nair and Joseph [24] could differentiate patients with congenital stationary night blindness, rod–cone dystrophy and central retinal occlusion from healthy controls using wavelet analysis of the full-field ERG. Discrete wavelet transform (DWT) analysis of the ERG waveform has been reported to be superior to the traditional time-domain analysis [21, 25]. Also, Gauvin et al. [25] investigated the luminance dependence of the full-field ERG response applying various DWT descriptors and introducing the use of the Hölder exponent. Lately, he applied DWT to assess the contribution of the oscillatory potentials to the full-field ERG [26]. With specific focus in glaucoma Miguel-Jiménez et al. [27] applied different types of wavelet analysis to the global flash mfERG response in glaucoma and found that CWT analysis, discrete wavelet transform (DWT) [28] and discrete wavelet packet transform [29] can separate advanced glaucoma from control and give additional information to the defect seen on Humphrey visual fields from these patients.

The aim of this study was to apply the DWT analysis to the two-flash multifocal electroretinogram (2F-mfERG) responses in primary open-angle glaucoma (POAG), in order to identify, among different mother wavelets, the most suitable one for the 2F-mfERG signal shape and to test its performance to differentiate glaucoma from control. In addition, we evaluated how the DWT analysis compares to the root-mean-square (RMS) calculations previously used in our studies for the analysis of the 2F-mfERG. For the most sensitive descriptor of the DWT, we then investigated the association with the ganglion cell–inner plexiform layer (GCIPL) thickness measured on OCT, as well as the visual field sensitivity using a customized visual field pattern based on the stimulus grid of the mfERG (mf103-pattern).

## Materials and methods

The study protocol was approved by the Ethics Committee of the University of Basel. All procedures followed the tenets of the Declaration of Helsinki. Informed consent was signed before participation.

Thirty-five healthy individuals and 25 POAG patients were included in this study. Inclusion criteria for all individuals included: visual acuity of 0.8 or better and refractive error between ±6 diopters of hyperopia or myopia. All patients were recruited at their regular glaucoma specialist consultation. Patients diagnosed with glaucoma presented glaucomatous optic neuropathy at fundus examination, significant localized thinning of the neuroretinal rim on the RNFL thickness map and, for POAG patients, a reproducible visual field defect (minimum three tests, Octopus 101, G2 protocol). Normal parameters in Octopus visual fields are: mean defect (MD) under 2.2 dB and a square root loss of variance (sLV) under 2.5 dB. Pre-perimetric glaucoma (PPG) patients presented with glaucomatous optic nerve characteristics associated with significant thinning of the RNFL on OCT, but no visual field defect.

Individuals diagnosed with systemic diseases which may affect the eye (e.g., diabetes), with regular use of medications that can influence retinal cell electrical activity (e.g., antidepressant, chloroquine, anticonvulsants) or who had previous ocular surgeries (e.g., cataract extraction, glaucoma surgery), were excluded.

All participants underwent visual acuity testing, slit lamp, fundus examination and tonometry (Goldmann). In addition a 2F-mfERG was performed to assess DWT and to decide on the best mfERG DWT descriptor to separate glaucoma patients from normal subjects.

**Fig. 1:**
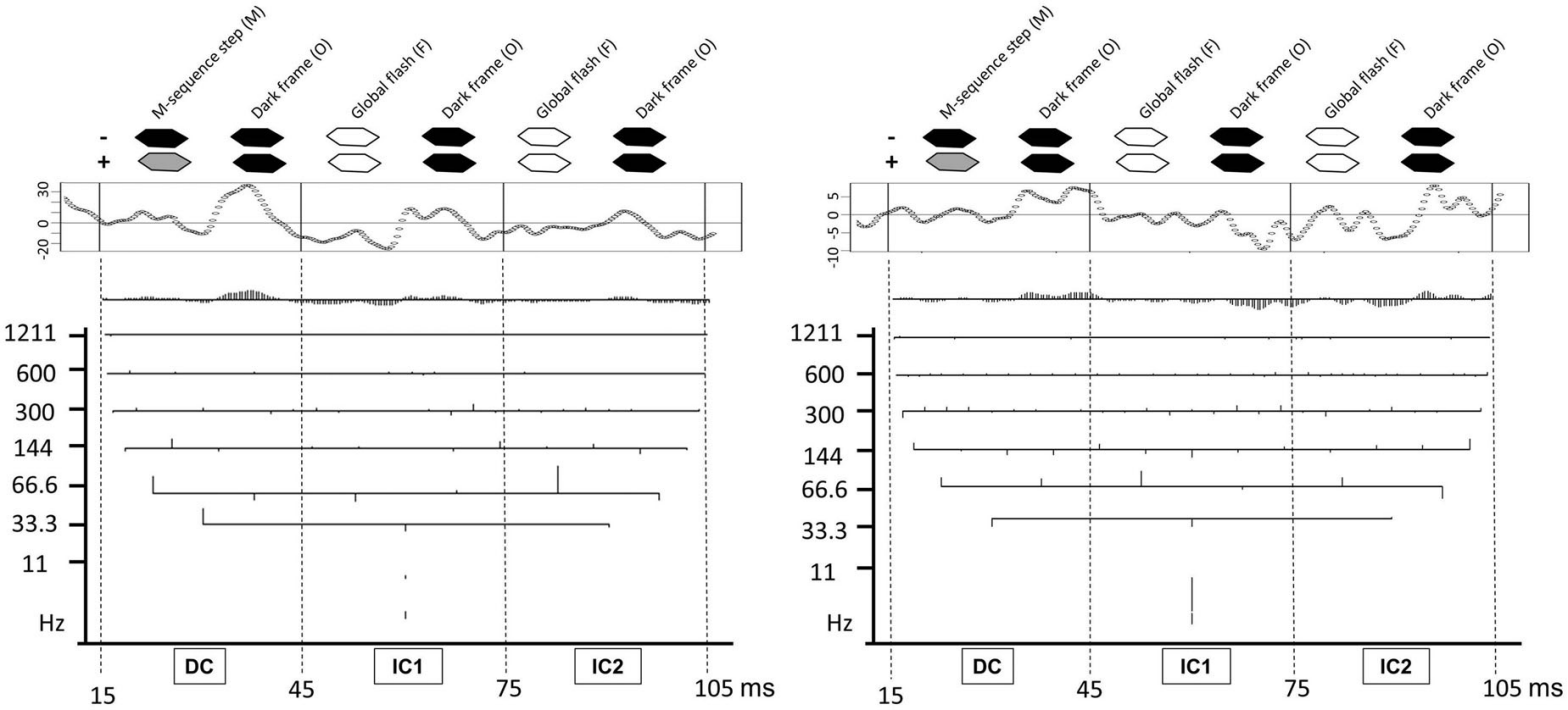
DWT analysis applied to the mfERG response from a control (*left*) and a patient (*right*). *Top* graphical representation of the 2F-mfERG M-sequence used here (MOFOFO), with frames displaced in time in order to better correspond visually to the recorded response. The original signal from one hexagon of the mfERG (waveform inside box on *top*) can be decomposed into many frequency levels, depending on the length of the time series. The first level (1211 Hz) corresponds to high frequencies (noise), while the highest level (11 Hz) corresponds to the lowest frequencies. For each frequency level, the *vertical lines* represent individual wavelet coefficients. For each level, the variance between these coefficients is computed and subjected to further analysis as the WVA (wavelet variance). Legend: *DC* direct component; *IC1* first induced component; *IC2* second induced component

The 2F-mfERG protocol used in this study has been described previously [3, 6, 30]. Briefly, it was recorded with VERIS Science 6.06, FMSIII (Electro-Diagnostic Imaging, USA). Pupils were dilated with a solution (Tropicamide 0.5%, Phenylephrine 1%, Spital Pharmazie USB, Switzerland), and a Burian Allen bipolar contact lens was used after application of a gel interface (Methocel 2%, OmniVision AG, Switzerland). The 103 hexagons stimulated the central 50° of the retina according to an m-sequence of length 2^13^ − 1 (*L*_max_ 100 cd/m^2^, *L*_min_ < 1 cd/m^2^). Each *m*-sequence step (*M*) was followed by two global flashes of 200 cd/m^2^ (*F*) at an interval of 26 ms. This interval was created by interposing dark frames at <1 cd/m^2^ (O), thus creating the stimulation sequence MOFOFO as shown in Fig. 1. We used a bandpass filter of 1–300 Hz. Total recording time was 10 min and 55 s, divided into 16 segments. Recordings with poor signal or contaminated by ocular movements were discarded and re-recorded. Artifact rejection filtering incorporated in the VERIS software was applied twice, as suggested by the manufacturer. Spatial averaging was not applied. The addition of a global flash to the standard mfERG m-sequence stimulus increases the contribution from the inner retina to the electrophysiological response [31] which is thought to enhance glaucoma detection [32, 33]. For the waveform analysis, cross-correlation between the m-sequence and the raw data recorded allowed individual focal waveforms to be derived. These were exported from VERIS using the command “export analyzed data.” For the DWT, the first-order 2F-mfERG response was analyzed from 15 to 105 ms for each focal response within the central 15° of the retina (19 responses).

In order to compare our results from the DWT to our results from previous studies, we also analyzed the RMS of the 19 central responses (15°) filtered at 1–200 Hz as this has shown best differentiation between POAG and control [2, 3, 30]. For this comparison, we analyzed the response to the m-sequence step (MOFOFO), the direct component, DC, at 15–45 ms and two induced components IC1 at 45–75 ms and IC2 at 75–105 ms (Fig. 1).

### Discrete wavelet analysis

In general, DWT represents discrete time series such as biosignals as real-valued functions of time and temporal frequency. An initial wavelet template, the “mother wavelet,” is changed in scaling (temporal frequency) and location (time). Changing width and location of the template creates a wavelet family that is correlated with the signal. The values of these correlations are used as coefficients to characterize the signal in frequency and time. Coefficients can be allocated to decomposition levels of descending frequency levels (high to low). The number of frequency levels depends on the length of the time series. Further technical information can be found in references [25, 26, 34].

Figure 1 shows an example of DWT analysis applied to a recorded signal from a control and a patient in this study. The original signal from one hexagon of the mfERG (waveform in the top box) can be decomposed into many frequency levels, depending on the length of the time series. The first level (1211 Hz) corresponds to high frequencies (noise), while the highest level (11 Hz) corresponds to the lowest frequencies. For each frequency level, the vertical lines represent individual wavelet coefficients. For each frequency level, the variance between these coefficients is computed and subjected to further analysis as the wavelet variance analysis (WVA).

Review of the literature showed that several mother wavelets have been applied in DWT of the ERG response, such as the “Daubechies” wavelets [20, 21, 25], the “Haar” wavelet [24, 35] and the “Mexican hat” [23, 36, 37]. For glaucoma, Miguel-Jiménez et al. have successfully applied DWT to the global flash mfERG response in advanced glaucoma. They analyzed a number of mother templates (not specified) and on visual comparison found the mother template Bior 3.1 to have the best performance [29]. In a later paper, they applied continuous wavelet transform using the Morlet waveform [27] with good results. In our study, we first compared the performance of different possible mother wavelets, such as the “Haar” wavelet and Daubechies S6, S8 and S10. Wavelet form Bior 3.1 was not contained in our software package and thus not tried. Performance was quantified as the statistical difference based on *p* values from mixed effects models. “Haar” wavelet, “Daubechies S6” and “Daubechies S10” showed larger *p* values comparing glaucoma against the control group. Larger *p* values are conventionally less significant when considering acceptance of a null hypothesis. Thus, decomposition was done using the “Daubechies S8” wavelet, which is default in the applied software package.

In the present study, seven frequency decomposition levels (1211–11 Hz) were evaluated.

In order to discriminate between study groups [controls and POAG (high-tension glaucoma (HTG), normal-tension glaucoma (NTG) and PPG)], various descriptors (e.g., describing factors: variance, energy, median, min, max IQR) were derived based on the coefficients. Here the best descriptor was the WVA which is in agreement with Gauvin et al. [25], who demonstrated the advantages of WVA application when using DWT (Daubechies wavelet) in ERG.

**Fig. 2:**
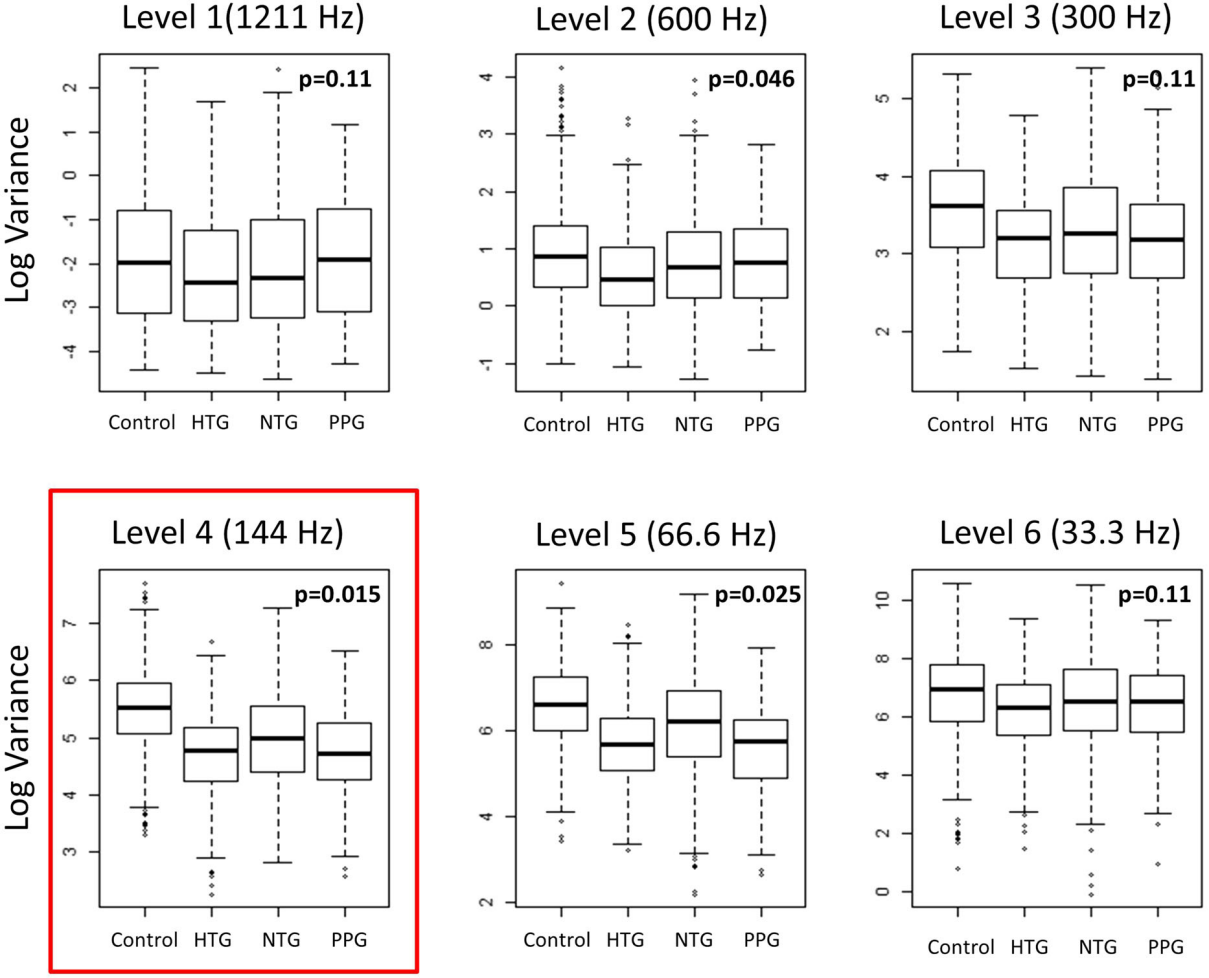
This figure summarizes the results of our decomposition. For each group, the *box plots* show the distribution of the wavelet variance (WVA, see Fig. 1) considering each location (19 focal mfERG waveforms) for each subject within each frequency level analyzed. Variance at frequency level 4, that is at 144 Hz, was the most sensitive distinguishing parameter (*p* = 0.015, *red box*). Legend: *PPG* pre-perimetric glaucoma; *NTG* normal-tension glaucoma; *HTG* high-tension glaucoma

Figure 2 summarizes our decomposition results. For each group, the box plots show the distribution of the WVA considering each location (19 focal mfERG waveforms) for each subject within each frequency level analyzed. Variance at frequency level 4 (144 Hz) was the most sensitive distinguishing parameter (*p* = 0.015, red box). Thus, we focused our subsequent analysis on WVA at frequency level 4 (WVA-144). In order to reduce the effect of potential outliers based on edge effects, WVA was explored on a log scale.

### Structure function analysis

**Fig. 3:**
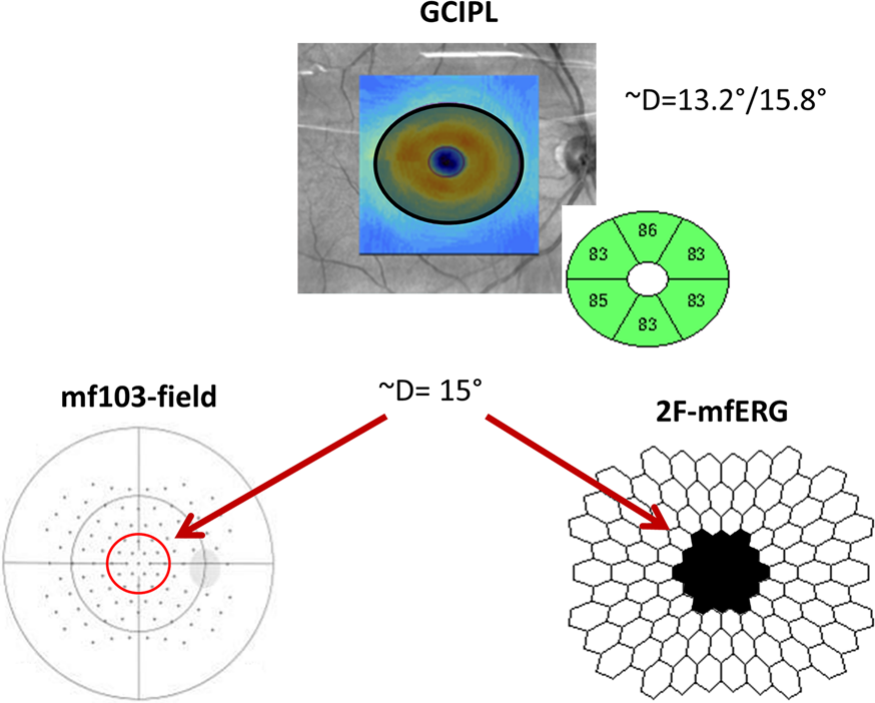
Graphical representation of corresponding areas from each examination (GCIPL, mf103-pattern and 2F-mfERG) compared in the study. Legends: *D* diameter; *GCIPL* ganglion cell–inner plexiform layer; *m103-pattern* Octopus pattern with 103 stimulus points; *2F-mfERG* double-flash multifocal electroretinogram

In a subgroup of 31 subjects (16 POAG, 15 controls), 2F-mfERG, OCT and customized visual fields could be obtained on the same day. In these subjects, we investigated the association between the most sensitive parameter of the DWT (DWT descriptor) and the GCIPL thickness as well as the visual field sensitivity. From each of these diagnostic exams, we analyzed the central 15° degrees of the retina (central 19 points, Fig. 3), as this is the area covered by the ganglion cell analysis in the OCT (4.0 mm vertical and 4.8 mm horizontal diameter). This area is also known to comprise the highest density of ganglion cells in the retina [38] and is the area previously reported as most sensitive in the 2F-mfERG in glaucoma [3].

### GCIPL analysis

GCIPL analysis was done using Cirrus SD-OCT [Carl Zeiss, USA, macular cube protocol (512 × 128)]. GCIPL thickness was calculated directly by the Cirrus software (version 6.5.0.722).

### Customized automated perimetry pattern

The customized automated perimetry pattern, mf103-pattern, (poster ARVO 2015: Invest. Ophthalmol Vis Sci 2015; 56(7):629) is implemented in the Octopus Perimeter (Octopus 900, Haag-Streit, Switzerland) using the same test parameters as in SAP (size III; 100-ms duration; background 10 cd/m2; 0 dB equaled to 4000 asb). Its main difference to SAP is that each stimulus presented to the patient is positioned to correspond to the center of each hexagon in the mfERG grid (103 stimulus locations covering 50°). Therefore, the sensitivity of each point from the mf103-pattern relates directly to the same hexagon from the 2F-mfERG response. Sensitivity values from each location were calculated individually in decibels transformed into linear units and compared either individually or averaged for the entire 15°.

### Statistical analysis

As WVAs have a skewed distribution, they were log-transformed in order to achieve an approximately normal distribution.

Comparisons between study groups were done using linear mixed effects models using age and gender as adjusting covariates. The mixed effects model generates averages over the 19 central focal WVA-144 values (144 Hz) for each subject. These averages are then compared between the study groups. Results are presented as differences of mean WVAs between study groups with corresponding 95% confidence intervals and *p* values. A *p* value <0.05 is considered significant. All evaluations were done using the statistical software R version 3.3.0 [39]. Wavelet analysis was performed using the package “wmtsa” contained in software R. Details are described by Walden [34].

ROC curves with corresponding AUCs were calculated to compare the diagnostic performance between different parameters and parameter combinations. Sensitivities and specificities were not calculated as this should only be done in large validated studies, which is not the case here [40]. In order to prevent overfitting due to small sample sizes, AUCs were calculated using penalized logistic regression with internal tenfold cross-validation (repeated 10 times) using the package “glmnet.” Penalized regression shrinks the regression coefficients, which prevents overoptimistic results based on small sample sizes. This calculation and comparisons between AUCs were done within the package “caret,” which is a convenient tool to examine predictive performances of statistical models. Within this package, the AUCs were internally 3 × 10-fold cross-validated and all pairwise differences were computed and tested to assess whether the difference is equal to zero [41, 42].

Calculations were examined with age-matched disease versus control groups to avoid the expected strong age influence on disease diagnosis which could mask the influence of study parameters. Matching was done based on propensity scores implemented in the package “nonrandom.”

## Results

Demographics from the analysis group are listed in Table 1. Average overall MD was 3.6 dB for POAG and 0.1 dB for control (*p* < 0.01). All patients presented with a controlled intraocular pressure, under topical medication if needed. In all statistical calculations, data were adjusted for age and gender.

**Table 1:** Demographic results from DWT analysis group

Group	Controls (*n* = 35)	PPG (*n* = 7)	NTG (*n* = 12)	HTG (*n* = 6)	*p* value (anova)
Age (years) Mean ± SD	50.8 ±12.1	63.8 ±14.7	60.1 ±13.7	62 ±10.2	0.017*
Gender (M/F)	11/24	6/1	9/3	5/1	
BCVA (decimal) Mean ± SD	1.0 ±0.1	0.99 ±0.13	0.96 ±0.06	0.91 ±0.13	0.035*
Refraction (mean 95% CI)					
Diopters	−0.6 −1.3/0.1	−0.4 −1.3/0.4	−0.02 −1.5/1.4	0.5 −1.4/2.6	0.558
Cylinder	−0.9 −1.1/−0.7	−0.6 −1.2/0.04	−0.6 −0.8/−0.4	−1.2 −2.1/−0.3	0.188
IOP (mmHg) Mean ± SD	14.2 ±2.9	13.4 ±1.5	12.8 ±1.7	12 ±3.7	0.066
CD Mean ± SD	0.3 ±0.07	0.7 ±0.1	0.7 ±0.1	0.7 ±0.1	0.000*

*SD* standard deviation; *PPG* pre-perimetric glaucoma; *NTG* normal-tension glaucoma; *HTG* high-tension glaucoma; *BVCA* best corrected visual acuity; *95% CI* 95% confidence interval; *IOP* intraocular pressure; *CD* cup-to-disk ratio; * *p* value <0.05 was considered statistically significant

### Discrete wavelet analysis

DWT analysis was applied to the 2F-mfERG, and the “Daubechies S8” wavelet was identified as the mother wavelet with the best performance for this dataset (see methods). Starting with the “Daubechies S8” wavelet, we looked for the best descriptor to differentiate glaucoma from controls by analyzing the variance of the wavelet coefficients at different frequency levels. Significant differences between control and POAG were seen at a frequency of 144 Hz (*p* = 0.015), 66.6 Hz (*p* = 0.025) and 600 Hz (*p* = 0.046) (Fig. 2). In our further analysis, we used the most sensitive descriptor we found, WVA-144, which corresponds to the decomposition level of 144 Hz.

In addition, we also analyzed WVA-144 in the individual epochs, DC, IC1 and IC2 but did not find a higher significance than seen in the DWT of the overall waveform between 15 and 105 ms.

We compared the predictive diagnostic performance of WVA-144 to RMS using the AUC of the ROC curve in an age-matched subgroup of 20 controls and 20 POAG patients. In these patients, RMS showed a significant positive correlation with WVA-144 (DC: *p* < 0.001, IC1: *p* < 0.001, IC2: *p* < 0.001). In the ROC analysis, WVA-144 and DC had the highest AUC values of 0.692 and 0.650, respectively. Both RMS-based AUC and WVA-144-based AUC could differentiate POAG from control. However, the DeLong test [43] demonstrated a statistical significant difference between the RMS-based AUC at each of the epochs (DC, IC1 and IC2) and the WVA-144-based AUC (*p* < 0.001). This suggests that the WVA-144 was more sensitive than the RMS measures, as it has the highest AUC.

### Structure–function analysis

In 15 controls and 16 POAG patients, 2F-mfERG, mf103-pattern and macular OCTs were obtained on the same day in order to investigate structure–function relationships.

**Fig. 4:**
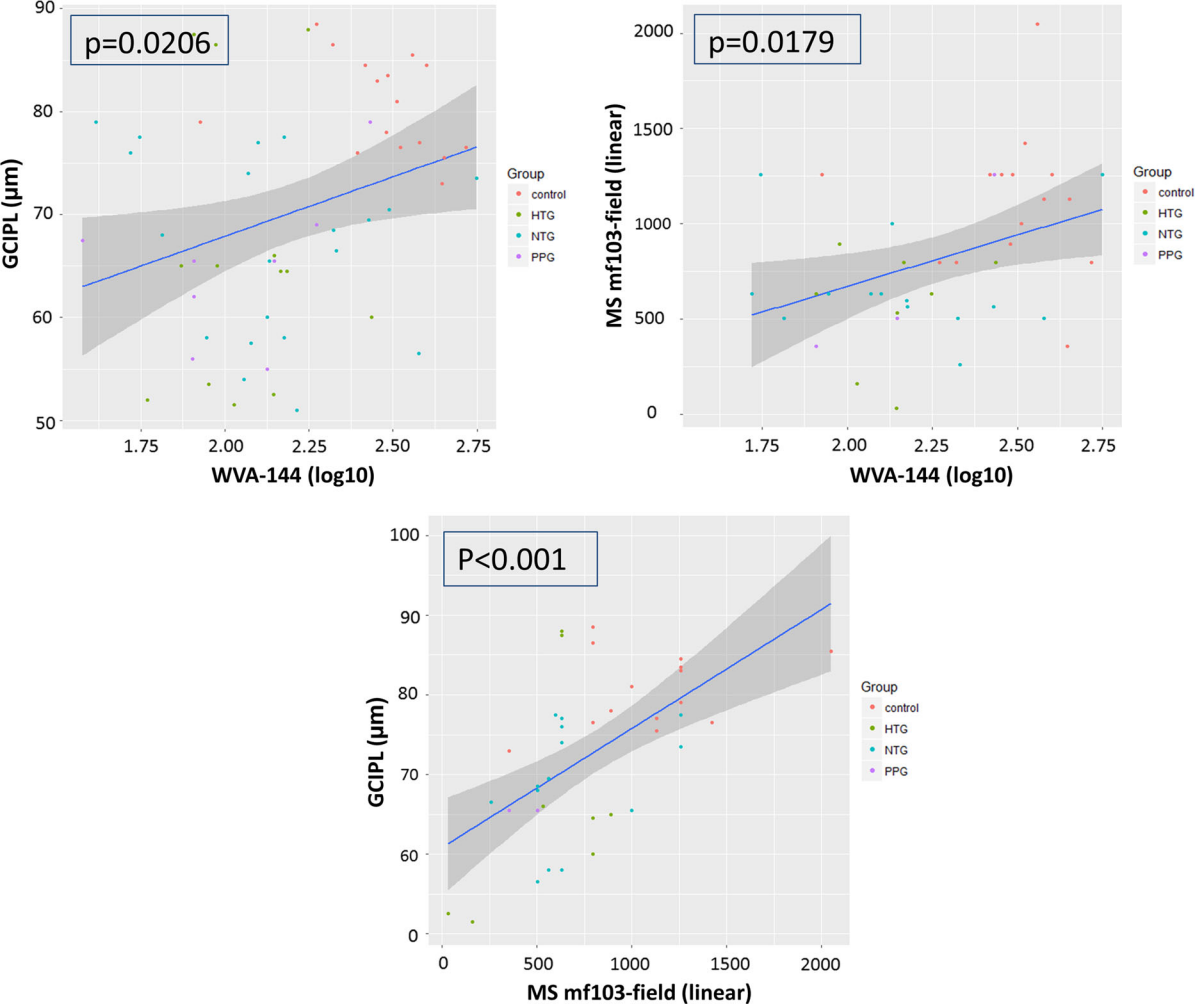
Graphical representation of structure–function relationship between each examination (GCIPL, MS and WVA-144) and its respective *p* values. Legend: *GCIPL* ganglion cell–inner plexiform layer in µm; *WVA-144* wavelet variance analysis 144 Hz (in logarithm); *MS mf103-field* mean sensitivity from mf103-pattern exclude “protocol” in linear values

Table 2 shows the characteristics of this population: Total MD was 4.1 dB in the glaucoma group and 0.5 dB in controls (*p* < 0.01). GCIPL was thinner in POAG [68.0 µm (±8.7)] than in controls (80.9 µm (±4.8), *p* < 0.01). WVA-144 was significantly lower in POAG than in controls (*p* = 0.038). Both GCIPL thickness and visual field linear sensitivity showed a significant positive relationship with WVA-144 and also between GCIPL and the mf103-pattern (Fig. 4).

**Table 2:** Demographic results from the structure–function analysis group

Group	Controls (*n* = 15)	PPG (*n* = 2)	NTG (*n* = 9)	HTG (*n* = 5)	*p* value
Age (years) Mean ± SD	49.3 ± 7.3	53.0 ± 25.5	61.1 ± 16.5	61.0 ± 6.3	0.102
Gender (M/F)	4/11	1/1	6/3	4/1	
MD (dB) Mean (95% CI)	0.12 (−0.8/1.0)	1.7 (3.0/5.9)	3.4 (2.2/5.4)	5.5 (3.8/17.3)	0.007*
MD 15° Mean (95% CI)	−0.07 (−1.1/0.5)	1.8 (0.6/2.2)	1.5 (0.6/2.2)	3.6 (1.0/3.7)	0.028*
GCIPL (µm) Mean ± SD	80.9 ±4.8	82.8 ±5.1	68.4 ±6.4	61.2 ±5.2	0.000*
WVA-144	323 ±112	164 ± 97.1	184 ± 141	145 ± 60.4	0.038*

Legend: *SD* standard deviation; *PPG* pre-perimetric glaucoma; *NTG* normal-tension glaucoma; *HTG* high-tension glaucoma; *95% CI* 95% confidence interval; *MD* overall mean defect in decibels; *MD 15°* average mean defect from the central 15°; *GCIPL* ganglion cell–inner plexiform layer in µm; *WVA-144* wavelet variance analysis frequency level 144 Hz; * *p* value <0.05 was considered statistically significant

For the individual types of glaucoma, Table 3 demonstrates the consistent significant group differences seen in WVA-144 and in GCIPL thickness, but not in the central mf103-pattern sensitivity. Within the central 15° (19 points), 56.2% of POAG patients had no defect, while 25% (*n* = 4) had 3 or more points with *p* < 0.05, the remainder showing only 1 or 2 individual points of probability <0.05.

**Table 3:** Mixed effects model results from group comparison between WVA-144, GCIPL and MS

	Difference of means	95% CI	*p* value
WVA-144			
PPG	−0.28	−0.52/−0.05	0.0180
HTG	−0.31	−0.50/−0.12	0.0018
NTG	−0.19	−0.37/0.00	0.0460
GCIPL			
PPG	−10.23	−18.9/−1.5	0.0220
HTG	−11.05	−18.0/−4.0	0.0026
NTG	−9.76	−16.5/−2.9	0.0058
MS			
PPG	−154.6	−586.4/276.9	0.4713
HTG	−217.5	−520.3/85.3	0.4951
NTG	−96.6	−381.2/188.0	0.1536

Results are presented as differences of means between study groups with corresponding 95% confidence intervals (95% CI) and *p* values

For the 95% CI, the 2.5th percentile and the 97.5th percentile are given

*WVA-144* wavelet variance analysis frequency level 144 Hz (transformed into logarithm units); *GCIPL* ganglion cell–inner plexiform layer (µm); *MS* mean sensitivity (decibels transformed into linear units)

**Fig. 5:**
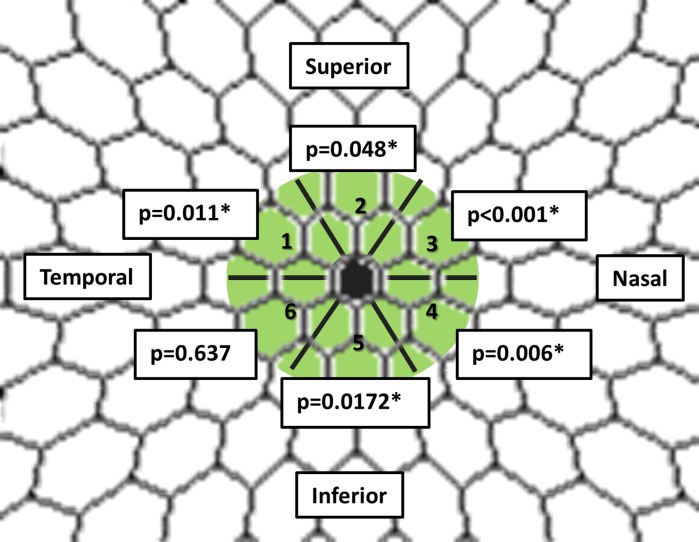
For the central 15 degrees, this figure shows the relationship between GCIPL thickness map sectors and the 2F-mfERG responses to the corresponding hexagons. *Green*: GCIPL = ganglion cell–inner plexiform layer thickness map (*retina view*). *p values <0.05 were considered significant. Calculations were adjusted for age and gender

Analyzing the data on a more focal basis, that is correlating individual GCIPL sectors from the thickness map to the corresponding hexagons from the mf103-pattern and 2F-mfERG, we found a statistically significant relationship between most of GCIPL sectors and WVA-144, with exception of the most temporal inferior sector (Fig. 5).

As glaucoma incidence increases with age, we compared diagnostic performance in an age-matched subgroup based on the mean of tenfold cross-validated penalized logistic regression.

The mean AUCs of Age, GCIPL, MS and WVA-144, as well as for all examinations combined, were calculated. Age had a small mean AUC of 0.44, and MS and WVA-144 had moderate AUCs (0.65, 0.64). GCIPL had the highest predictive performance (0.880), outperformed only by the combination of all exams together GCIPL + MS + WVA-144 (AUC: 0.945), showing that MS and WVA-144 contribute to glaucoma diagnosis with additional information.

## Discussion

In electrophysiology, electrical activity from retinal cells is recorded from the corneal surface. The resulting waveforms are traditionally analyzed in amplitude and latency (peak time) [44, 45]. In the global flash mfERG response, the root-mean-square (RMS) method is often applied [8, 46]. Decomposition of the mfERG signal into its time–frequency components, applying DWT analysis, offers an interesting objective and automated approach to further explore electrophysiological responses. In the full-field ERG, DWT has been shown to reveal additional contributions as shown by Gauvin et al. [26]. For example, Gauvin et al. [25, 47] were able to show a strong correlation between DWT descriptors and different parts of the full-field ERG such as the *a*-wave, the *b*-wave and also oscillatory potentials at 80 and at 160 Hz [26]. Thus, DWT offers a promising tool, in particular as the response to the global flash mfERG waveform does not show typical *a*- or *b*-waves due to the adaptive properties of the stimulus response.

In our study, we could differentiate patients with early, rather than advanced, POAG (including PPG) from controls using DWT analysis on the two-flash mfERG response. In the present study, we were able to directly compare electrophysiological findings not only to visual fields, but also to the OCT. When our DWT findings were compared to a waveform analysis that is more frequently applied by clinicians, the RMS [2, 3, 30], we found their performance to be similar, although DWT was statistically better in distinguishing POAG from control. WVA could be more powerful because RMS is based on overall variance, whereas WVA distinguishes between frequency levels. If there is more variance in a certain level, this could be detected in WVA, but blurred in RMS. Thus, the application of DWT to the 2F-mfERG is a valid additional diagnostic tool in glaucoma. It offers an objective approach to the electrical signal that takes into consideration not only time–frequency but also variance within the response. This is an advantage over time-domain analysis, where variance of amplitude can be analyzed at different frequencies following bandpass filtering.

In our population, DWT analysis of the two-flash mfERG using Daubechies S8 was able to differentiate glaucoma from control in OAG with an AUC of 0.64. While sensitivity and specificity appear lower than reported by Miguel-Jiménez et al., our analysis was restricted to the central retina in all subjects included. In addition, our patient group consisted of primarily early glaucoma with an overall average MD of 3.6 dB. Within the central 15° analyzed, mean MD was 2.7 dB in HTG, 1.3 dB in NTG and 1.0 dB in PPG (Table 2, [48]). In advanced glaucoma, Miguel-Jiménez et al. [28] were able to identify signal characteristics in a two-flash mfERG that could differentiate OAG from control (DWT, mother wavelet Bior 3.1). In addition to the 50 patients analyzed, they demonstrated a good topographic correlation between the two-flash mfERG DWT findings and the Humphrey fields in two patients with advanced OAG [28]. In a subsequent paper, using discrete wavelet packet decomposition analysis the same group focused on the induced component (60–90 ms) of their global flash mfERG. Analysis of the visual field sectors affected by glaucoma in 25 OAG patients found a sensitivity of 0.81 and a specificity of 0.73 [29]. Further, using CWT (Morlet waveform) in OAG and healthy subjects, sensitivity could be increased to 0.894 and specificity to 0.844, again only including sectors identified as glaucomatous and again looking at the induced component [27].

Some of the differences between our findings and those of Miguel-Jiménez et al. [27–29] may be related not only to the different glaucoma stages analyzed, but also to the different selection of areas analyzed: only areas with field defect versus the central 15°, irrespective of the location of the field defect. Differences may also result from the use of different visual field patterns. In contrast to Miguel-Jiménez et al. [27–29], we did not average hexagons to fit single visual field locations. Our customized visual field pattern (mf103-pattern) allowed direct spatial correlation between visual field sensitivity and the mfERG response.

In our early POAG patients, the WVA-144 was able to identify glaucomatous alterations in the central 15° even when the visual field could not. Similar findings have been observed in outer retinal disease, such as retinitis pigmentosa [49]. Our findings are in agreement with Takagi et al. [1], who suggested that a more sensitive functional test should be able to detect glaucoma earlier than the visual field as GCIPL thickness may be already compromised in glaucoma even when no defect is identified by standard automated perimetry.

Structure function investigation showed a significant positive relationship between the WVA-144 from the 2F-mfERG and the GCIPL, between the WVA-144 and the mf103-pattern, and also between GCIPL and the mf103-pattern. A detailed analysis demonstrated a significant relationship between WVA-144 and the GCIPL thickness map sectors except for the temporal inferior sector. It has been demonstrated that superior visual field defects (inferior retina) are more common in glaucoma than inferior field defects [50–52]. In our POAG patients, there was no predominance of superior or inferior field defects. The lack of correlation in the temporal inferior quadrant is an interesting finding. Follow-up studies in a larger population are needed to assess whether that is an incidental finding.

AUC analysis with age-matched groups demonstrated that GCIPL alone had a high predictive performance, which improved further when MS and WVA-144 were added. Thus, we agree with a recent editorial from the American Academy of Ophthalmology, in which Medeiros and Tatham [53] suggest that for a correct early diagnosis it is better to combine different examinations rather than to keep searching for a single parameter.

Here, the most significant difference between POAG and controls was localized at 144 HZ, which coincides with the frequency range of the oscillatory potentials (OPs) [54]. OPs of full field are thought to reflect activity between bipolar, amacrine and ganglion cells and are generated within the IPL [54, 55]. In experimental glaucoma, swelling of ganglion cell dendrites in the IPL has been described as an earliest manifestation of glaucoma [56]. Thus, changes at the OP frequency level may be expected in glaucoma.

Indeed, OPs from the mfERG have previously been described as a sensitive measure of glaucomatous dysfunction, especially with use of a slow sequence mfERG [32, 57]. In the monkey and in humans, Rangaswamy et al. [57] found glaucoma-induced changes in fast OPs at about 143 Hz in all areas studied, even when field defects were only moderate (MD between −5 and −10 dB). These fast OPs showed a good positive correlation to the estimated ganglion cell density. Slow OPs at about 77 Hz were affected primarily in the center, when MD was larger than—10 dB [57]. In primates with experimental glaucoma, a two-flash mfERG (MOFOFO) showed changes primarily in a low-frequency component contributing to DC/IC1 but also in a high-frequency component at a little below 150 Hz [8].

Fortune et al. [58] could show that OPs in glaucoma are diminished in the first induced component of the one global flash mfERG, while in the two-flash mfERG Palmowski-Wolfe et al. [59] showed these to be primarily affected in IC2. Compared to standard mfERG, OPs from the global flash mfERG are more sensitive to detect glaucomatous dysfunction in human and experimental glaucoma [32].

In future studies, the performance of the DWT may be increased further by including various descriptors in the analysis in addition to only studying the most sensitive descriptor. We have not addressed the possibility that different descriptors are affected differently in HTG, NTG and PPG. Assessing ratios between different decomposition levels could reduce outside influences (such as noise) and thereby the coefficient of variation. This may further improve separation between patient groups and control. Another improvement might be to include the Hölder exponent, as proposed by Gauvin [25, 49] which characterizes the complexity of the waveform and was able to identify electrophysiological changes in the ERG prior to changes in the visual field in retinitis pigmentosa [49]. In addition, the use of scalograms [24, 25, 47] that summarize the particular contribution of individual coefficients over time may improve understanding of disease.

To date, electrophysiological measures are not incorporated in the clinical routine diagnosis of glaucoma, although they have been shown to be sensitive in this disease. This may change with the automatization of DWT analysis. DWT offers an exciting new approach with the potential of development of automatic analysis procedures and with visualization of results (e.g., scalograms) and thus facilitation of interpretation by clinicians.

A limitation in our study could be the imbalance in age between glaucoma and control groups. Therefore, all calculations were adjusted for age and AUC calculations were performed in perfectly age-matched groups. Another imbalance was seen in the gender distribution which we took into account in our statistical analysis. Nonetheless, previous studies on GCIPL could not identify gender differences in this retinal layer [60] (GCIPL). Cohn et al. observed that in the visual field, sensitivity of the left and right hemifield may differ in females but not males. When sensitivity values were averaged over the field, as done in our study, no gender differences were found [61].

In conclusion, we demonstrated that the application of wavelet analysis to the 2F-mfERG recordings improve glaucoma diagnosis when added to GCIPL and visual field analysis. It may improve follow-up as significant differences were seen between control and glaucoma but not in the visual fields when results were adjusted for age and gender. This confirms suggestions of early central retinal structure and function involvement even prior to central field defects.
